# Expression of concern: Flavin-adenine-dinucleotide gold complex nanoparticles: chemical modeling design, physico-chemical assessment and perspectives in nanomedicine

**DOI:** 10.1039/d5na90056e

**Published:** 2025-08-07

**Authors:** Celia Arib, Nadia Bouchemal, Maria Barile, Didier Paleni, Nadia Djaker, Nathalie Dupont, Jolanda Spadavecchia

**Affiliations:** a CNRS, UMR 7244, CSPBAT, Laboratoire de Chimie, Structures et Propriétés de Biomatériaux et d'Agents Thérapeutiques, Université Paris 13, Sorbonne Paris Nord 1 Rue Chablis 93000 Bobigny France jolanda.spadavecchia@univ-paris13.fr; b Dept. of Biosciences, Biotechnology and Biopharmaceutics, University of Bari “Aldo Moro” Via Orabona 470126 Bari Italy; c BioEVEN Start-up 75 Rue de Lourmel 75015 Paris

## Abstract

Expression of concern for ‘Flavin-adenine-dinucleotide gold complex nanoparticles: chemical modeling design, physico-chemical assessment and perspectives in nanomedicine’ by Celia Arib *et al.*, *Nanoscale Adv.* 2021, **3**, 6144–6156, https://doi.org/10.1039/D1NA00444A.

The following article “Flavin-adenine-dinucleotide gold complex nanoparticles: chemical modeling design, physico-chemical assessment and perspectives in nanomedicine” by Celia Arib^a^, Nadia Bouchemal^a^, Maria Barile^b^, Didier Paleni^c^, Nadia Djaker^a^, Nathalie Dupont^a^ and Jolanda Spadavecchia*^a^ has been published in *Nanoscale Advances*.

*Nanoscale Advances* is publishing this expression of concern in order to alert our readers that following the publication of a correction to this paper to update panels B and B1 in Fig. S3 (https://doi.org/10.1039/D3NA90084C), additional concerns have been raised regarding the reliability of Fig. 5, S1 and S3. An investigation is underway, and Sorbonne Paris Nord University and the CNRS have been requested to investigate the concerns.

An expression of concern will continue to be associated with the article until a final outcome is reached.

Jeremy Allen

31^st^ July 2025

Executive Editor, *Nanoscale Advances*

